# Impacts of Psychosocial Stress and Age Acceleration on Cognitive Impairment: The Health and Retirement Study

**DOI:** 10.1093/geroni/igaf122.435

**Published:** 2025-12-31

**Authors:** Sarah Forrester

**Affiliations:** University of Massachusetts Chan Medical School, Worcester, Massachusetts, United States

## Abstract

Psychosocial stress affects physiological and cognitive outcomes. This study examines race and sex differences in psychosocial stressors and biological age acceleration (BAA) and how psychosocial factors and BAA impact cognitive impairment (CI). Data came from 2,083 participants in HRS who had biological (blood pressure, BMI, pulmonary function, glucose, CRP, and cholesterol), cognitive, and psychosocial data (chronic stress, wealth, lifetime trauma, depression symptoms) available 2006 – 2016. CI was measured with the Telephone interview for Cognitive Status (TICS; score <12 = CI). BAA was measured using the KDM equation as biological age minus chronological age. Participants were 66 years on average (SD = 8) at analytic baseline, majority White (76%), and female (60%). Linear mixed effects and frailty models were adjusted for age and education and adjusted for multiple testing. Black participants had higher CI, stress, and BAA. The BAA and depression relationship was stronger for men compared to women (B = 0.75, 95% CI: 0.32 – 1.19). Each year increase in BAA was associated with a significant hazard of incident CI (HR: 1.01, 95% CI: 1.01 – 1.02) and was stronger among women than men (HR: 1.02, 95% CI: 1.01 – 1.03). BAA was was associated with a lower hazard of CI when trauma was present (HR: 0.973, 95% CI: 0.96 – 0.99). Eighty-three percent of the association between chronic stress and incident CI was accounted for by BAA. BAA may be a mechanism through which stress impacts risk of CI and important for prevention efforts among those most affected.

